# Accumulation and toxicological effects of nonylphenol in tomato (*Solanum lycopersicum* L) plants

**DOI:** 10.1038/s41598-019-43550-7

**Published:** 2019-05-07

**Authors:** Lei Jiang, Yi Yang, Yong Zhang, Ying Liu, Bo Pan, Bingjie Wang, Yong Lin

**Affiliations:** 1 Environment and Plant Protection Institute, Chinese Academy of Tropical Agricultural Sciences, Haikou, 571101 China; 2 Key Laboratory of Integrated Pest Management on Tropical Crops, Ministry of Agriculture, Haikou, 571101 China; 3 Hainan Entry-Exit Inspection and Quarantine Bureau, Haikou, 570311 China

**Keywords:** Oxidoreductases, Abiotic, Environmental impact

## Abstract

Nonylphenol (NP) is one of the most worrisome and ubiquitous environmental endocrine disruptors. The tomato is one of the most important agricultural plants in the world. However, little is known about the toxicological effects of NP on tomato crops or the accommodative responses of tomato plants to NP stress. Thus, in this study, relevant tests were performed using pot experiments, and they indicated that when the NP concentration in the soil was elevated from 25 mg kg^−1^ to 400 mg kg^−1^, NP was progressively accumulated by the tomato plants. The NP induced growth inhibition and a declined in the total chlorophyll content, and it aggravated membrane lipid peroxidation in tomato plants. When confronted with NP stress, the tomato plants correspondingly induced their antioxidant enzymes via both molecular and protein pathways to relieve the NP-induced oxidative stress. All the above results would be illuminating for developing strategies to address NP-induced damage to agricultural output, food quality and public health.

## Introduction

Endocrine-disrupting chemicals (EDCs), which include a large number of native or artificial-compounds that are widely used in industry and daily life, have been shown to be causally linked to disturbed bioendogenous hormone activities (including the synthesis, release, transport, binding, action and removal of the hormones) in human beings or wildlife^1–4^. Many studies have shown that after their entry into the environment EDCs can seriously affect the ability of organisms to reproduce as well as engage in some other essential body functions (such as growth, cognition, aggression, communication, etc.) and in turn break the persistence and balance of the ecological system^3–6^. Notoriously, one of the most worrisome and ubiquitous EDCs in the environment is nonylphenol (NP). NP is primarily degraded from nonylphenol ethoxylates (NPEOs), which are applied extensively applied as surface-active agents during industrial production, daily life and agricultural practices^6,7^. In particular, NP is more poisonous and persistent than its parent compounds^8,9^.

Primarily due to the agricultural application of sewage sludge, wastewater irrigation and the use of agrochemicals containing NPEOs as additives, up to several hundred or even thousand mg/kg (dry weight) NP have been detected in soil and sediment samples^10,11^. As reported by Vogel *et al*.^12^ in their study on the migration ability of NP in soil, 730 days after application, up to 99% of the nonylphenol remained in the topsoil profile, which could then harm the crop yield and food safety through crop uptake and eventually pose a threat to human health by means of its enrichment and transmission within the food chain^10,13–16^. Hence, it is imperative to obtain knowledge about the eco-physiological effects of NP on crop growth and its mechanism. However, as far as we know, in recent decades, more and more attention has been paid to the broad-spectrum toxicity of NP to both invertebrates and vertebrates^1,16,17^, and only a handful of studies have been performed on the accumulation, degradation and toxicity of NP in crops, such as beans and wheat^18–20^.

The tomato occupies an important position in global vegetable production. However, investigation on the pollution status of phenolic substances in vegetables has shown that, after celery, tomato fruits ranked second among the surveyed vegetables regarding NP residue contents^21^. So far, although an increasing amount of research has addressed the effects of salt stress, water stress and pesticides on the antioxidant system of tomatoes^22–26^, much less is known concerning the adverse effects of soil NP on tomato crops and the accommodative response of the tomato plants to NP stress. Thus, in this paper, tests were performed using pot experiments to observe the biochemical, physiological and molecular responses of tomato (*Solanum lycopersicum* L) plants to NP-induced toxicity.

Several contaminants can be present in soil, such as pesticides, polycyclic aromatic hydrocarbons and heavy metals, which are ready to intrude into crop plants. Plants have evolved sophisticated defence mechanisms to counteract the stress induced by these toxicants^27,28^. The antioxidant system is a key constituent consisting of a variety of independent yet cooperating enzymes^29,30^. Given the above information, this paper was designed to (1) discern the responsive along with the counteracting mechanisms of tomato seedlings to NP stress, (2) explore competent indicators for the level of NP pollution in the soil-crop system, and (3) provide insight for further developing strategies to alleviate the NP-induced risk to crop yields, food safety and human health.

## Materials and Methods

### Materials

NP, a mixture of various isomers, was purchased from Dr. Ehrenstorfer GmbH, Germany, with a purity of >99%. Tomato seeds (*Solanum lycopersicum* L.) were supplied by the Academy of Agricultural Science in Hainan, China.

The undisturbed soils used in this study were collected from the experimental station at the Chinese Academy of Tropical Agricultural Sciences, in the city of Danzhou, Hainan province (N 19.50°; E 109.48°). The surface soil (0–20 cm) was collected, air-dried, manually crushed, and passed through a 3-mm sieve. Some of the physicochemical characteristics of the soil are listed in Table 1.

**Table 1:** Some physical and chemical properties of the soil tested.

Soil type^*a*^	pH	Organic carbon (%)	Texture			Total N (g kg^−1^)	Available P (mg kg^−1^)	Available K (mg kg^−1^)	CEC (cmol^(+)^ kg^−1^)
			sand (%)	silt (%)	clay (%)				
Eutric cambisols	6.27	0.65	40.1	36.3	23.6	1.07	13.7	41.9	25.68

^*a*^Soil type classification based on FAO-Unesco system.

### Plant culture and treatment

Uniform tomato seeds were soaked and disinfected in 5% sodium hypochlorite solution for 10 min, rinsed thoroughly and bathed in water in the dark at 27 °C on a culture dish for 24 h. Afterwards, the homogeneous budded seeds were seeded in 1 L plastic pots (each with 12 seeds) containing 1200 g of soil (with NP concentrations of 0, 25, 50, 100, 200, and 400 mg kg^−1^ soil) and were cultured in an artificial climate incubator (light intensity, 300 µmol m^−2^ s^−1^; a light/dark cycle of 14/10 h at 27/22 °C; humidity, 70%). The NP concentration used here was based on a realistic environmental pollution level^10,11^. The plants were allowed to grow for 28 days while a 70% relative water content was maintained in the soil by daily watering. The third true leaf on each plant was picked for the detection and analysis of the total chlorophyll, cellular lipid peroxides, activity of antioxidant enzymes, and transcript abundance.

### Growth parameters

The elongation of the taproots and the primary stems were measured. To measure the biomass, the newly collected samples were kept in an electro-thermostatic blast oven at 105 °C for 20 min, then 80 °C for 48 hours, and finally weighed with an electronic balance.

### Chlorophyll and lipid peroxidation

The total chlorophyll content was quantified according to Lichtenthaler and Buschmann^31^. Freshly collected leaves were cut into pieces, homogenized in 95% ethanol and centrifuged, and then, the absorbance values of the supernatant were detected at 663, 645 and 470 nm with a UV-visible spectrophotometer (SPECORD^®^ 200 Plus, Analytikjena, Germany).

The lipid peroxidation level in the plant tissues was characterized quantitatively using the method described by Ohkawa *et al*.^32^.

### Antioxidant enzyme activity

The superoxide dismutase (SOD, EC 1.15.1.1) activities were expressed in terms of their inhibitory capability, as the photoreduction of nitro blue tetrazolium (NBT) discussed in Dai *et al*.^33^. The peroxidase (POD, EC 1.11.1.7) activity was evaluated by detecting the enzyme’s ability to catalyse the oxidation of guaiacol by hydrogen peroxide^34^. The activity of the catalase (CAT, EC 1.11.1.6) was analysed using the method reported by Mckee *et al*.^35^. The ascorbate peroxidase (APX, EC 1.11.1.11) activity was determined by monitoring the rate of ascorbate oxidation caused by H_2_O_2_, as reported by Jiang *et al*.^36^. The activity of glutathione S-transferase (GST, EC 2.5.1.18) was detected in accordance with Habig *et al*.^37^. The activity of glutathione reductase (GR, EC 1.6.4.2) was investigated by observing the decline in the absorbance at 340 nm caused by the oxidation of NADPH as it participated in the reduction of oxidized glutathione^38^.

### Transcript abundance

The total RNA from the plant tissues was extracted with TRIzol reagent (Invitrogen, Carlsbad, CA) and reverse-transcribed into cDNA using a ProtoScript First Strand cDNA Synthesis Kit (NEB, Ipswich, MA). The primers for the genes *Cu/Zn-SOD*, *POD*, *CAT*, *APX*, *GST* and *GR* were designed using the GenBank database (NCBI). The oligonucleotide sequences of the primers used in this study are as follows: Cu/Zn-SOD: 5′-GGGTTGTCACTCTATCTCA-3′ (forward) and 5′-CACTATGTTTCCCAGGTC-3′ (reverse), POD: 5′-ATGCGGATAATCTCAAGT-3′ (forward) and 5′-AGTGGTCCATCTACAAGC-3′ (reverse), CAT: 5′-CATTCGCCTTCTTCTACG-3′ (forward) and 5′-GACAATATGTCCAGGGTTA-3′ (reverse), APX: 5′-CCATATCCGCCTCCACGAC-3′ (forward) and 5′-CAGCCCAAGCGAACCAAA-3′ (reverse), GST: 5′-TCCGTTTCTTCTACCTGATG-3′ (forward) and 5′-CATAGAATGCTTATACCAAG-3′ (reverse), and GR: 5′-CGTGGTTGCGTTCCTAAA-3′ (forward) and 5′-TGGACGATGAGCCCTACT-3′ (reverse). The PCR products were loaded onto a 1% (*w/v*) agarose gel, and they were separated by electrophoresis and stained with ethidium bromide^30^.

### NP extraction and analysis

In this study, to quantify detect the accumulation of NP in the tomato seedling tissues two grams of homogenized plant samples were ultrasonically extracted with 15.0 mL of methanol/dichloromethane (1:4, *v/v*). After centrifugation, the supernatant was purified and concentrated with a solid phase amino extraction (NH_2_ SPE) column and analysed using high-performance liquid chromatography with a fluorescence detector^39,40^. The spike recovery tests indicated that the recovery of the extraction procedure was 90.1–95.2% for nonylphenol when applied to plant samples, with a relative standard deviation (RSD) of 1.5%-2.3% (n = 5), and the detection limit was 0.008 mg kg^−1^.

### Statistical analysis

All the data were analysed using SPSS software version 19.0 for Windows. Each treatment was repeated three times with 10 seedlings each. Each of the presented data points were represented as the means ± standard deviation (SD). The significance of the differences among the differently treated groups was determined with a one-way analysis of variance (ANOVA), followed by Fisher’s least significant difference (LSD) method for pair-wise multiple comparisons. The statistical significance was set at *p* < 0.05.

## Results and Discussion

### Accumulation

**Figure 1:**
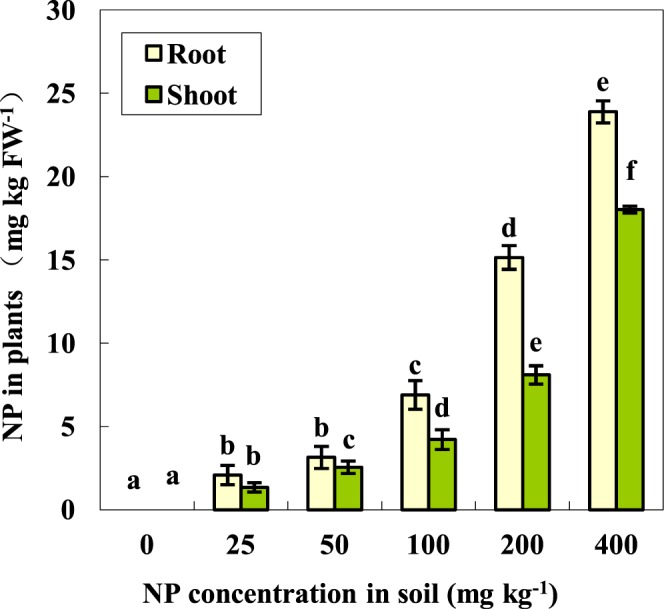
Accumulation of NP in the shoots and roots of the tomato seedlings (means ± SD, n = 3). Data with the same letter did not differ significantly at the 5% level.

To evaluate the potential NP uptake by plants from NP-contaminated soil, the NP contents in the aerial and underground parts of the tomato plants were analysed. As shown in Fig. 1, the NP contents in both the aboveground and underground parts of the seedlings kept increasing with the increased NP application rate in the soil. With exposure to 400 mg kg^−1^ NP for 28 days, the accumulation of NP in the shoots and roots reached up to 18.03 and 23.89 mg kg^−1^, respectively (Fig. 1). In addition, under the same conditions, the NP levels in the shoot tissues were relatively lower than those in the roots, suggesting that the NP would be a systemic organic chemical. As such, the NP could be absorbed from the soil by the roots and then partly transferred to the above-ground parts, and then ultimately located throughout the whole plants. Similar observations were previously reported in studies on the uptake and translocation of pesticides in plants^27,28^.

### Growth

**Figure 2:**
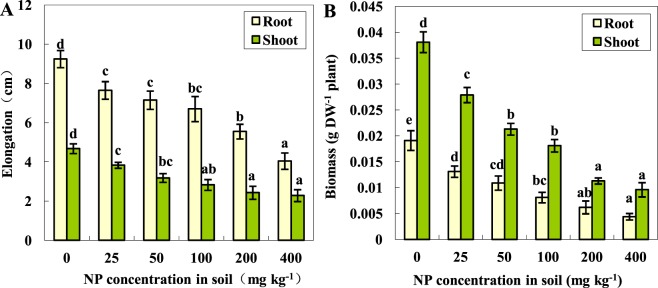
Effects of NP on the growth of the tomato seedlings (means ± SD, n = 3). Data with the same letter did not differ significantly at the 5% level.

As shown in Fig. 2, the deleterious effect of NP on tomato seedlings was visually reflected by the stunted plant growth. With the increase in the soil NP concentrations from 25 to 400 mg kg^−1^, the growth of the tomato plants exhibited a gradually declining trend. Compared to the control, a soil treatment of 400 mg kg^−1^ NP for 28 days markedly reduced the shoot lengths and root lengths by 50.1% and 56.3%, respectively, and they correspondingly decreased the dry weights of the shoots and roots by 74.8% and 77.0%, respectively. The results of the growth trials performed here indicated that the plant growth was notably affected by the presence of NP in the soil during the first four weeks, and tomato roots with a much stronger accumulation capacity for the NP in the soil appeared to be more susceptible to NP stress than the shoots.

### Chlorophyll

**Figure 3:**
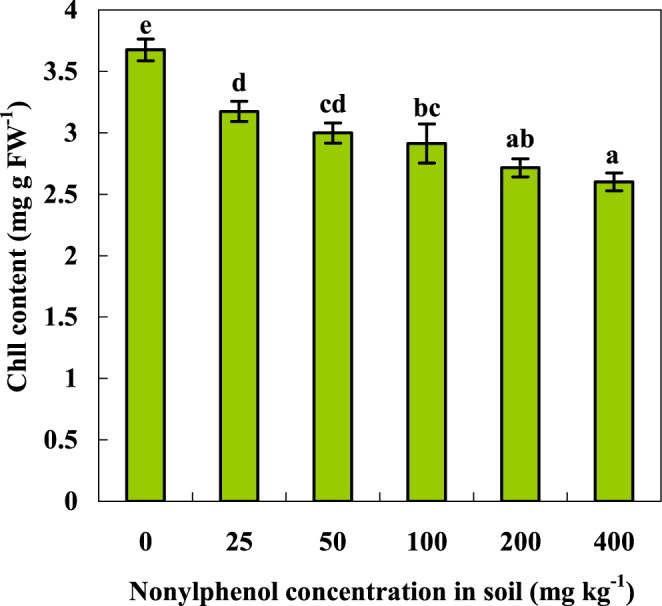
Effect of NP on the chlorophyll contents of the tomato seedlings (means ± SD, n = 3). Data with the same letter did not differ significantly at the 5% level.

Photosynthesis is the cornerstone of plant growth and development, and chlorophyll is the primary element associated with photosynthesis. Thus, it is essential to study the effect of NP on the chlorophyll contents of crop plants. As shown in Fig. 3, the chlorophyll contents of the tomato plants decreased progressively with the increasing NP concentration in the soil. Exposure to 25 mg kg^−1^ NP could downgrade the chlorophyll content of tomato seedlings by 13.7% compared to the control. The same pigment-bleaching phenomenon was previously reported in a study on the effect of BPA, another environmental endocrine disruptor, on physiological processes in plants^41,42^.

### TBARS

**Figure 4:**
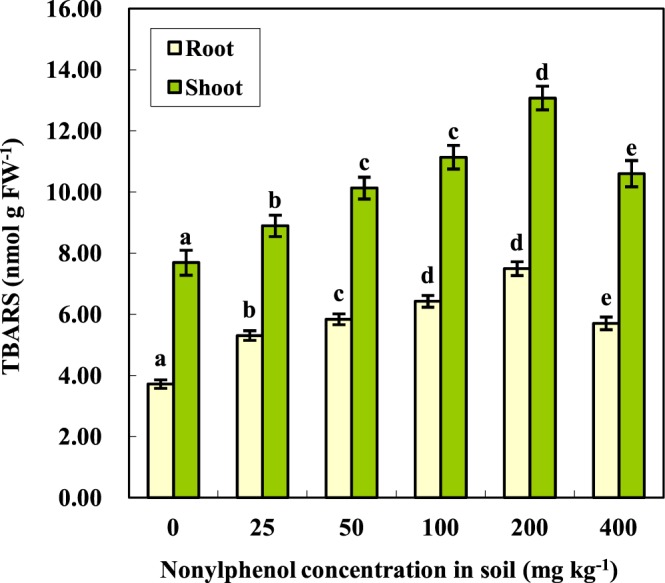
Effects of NP on the TBARS content in the tomato plants (means ± SD, n = 3). Data with the same letter did not differ significantly at the 5% level.

It is broadly accepted that there is a causal link between exposure to environmental stresses (such as organic contamination) and the aggravation of membrane lipid peroxidation in plants^43^. Thus, the content of thiobarbituric acid reactive substances (TBARS), an indicator of the membrane lipid peroxidation level, was determined in this study. Compared with the control, the TBARS contents of the tomato seedlings were significantly raised by NP stress (Fig. 4). In addition, the maximum values in both the shoots and roots appeared in the 200 mg kg^−1^ NP treatment, which were 1.70 and 2.02 times the control values, respectively. It is not difficult to observe that the roots, which accumulated more NP than the shoots, suffered more serious oxidative damage. The TBARS contents then failed to rise further as the NP concentration increased from 200 mg kg^−1^ to 400 mg kg^−1^. As shown in Fig. 4, at 400 mg kg^−1^ NP, the TBARS contents of the shoots and roots were 1.38 times and 1.53 times the control, as reduced by 18.9% and 23.9%, respectively, in comparison with the 200 mg kg^−1^ NP treatment. These results might be ascribed to the leakage of cellular contents caused by the destruction of cell structural integrity, which is causally associated with relatively higher levels of NP stress^30,36^.

### Enzyme activities

It has been broadly recognized that abiotic stress can destroy the cellular redox equilibrium and in turn bring about the overproduction and superabundant accumulation of reactive oxygen species (ROS)^27,44^. Now that oxidative stress induced by NP has resulted in pigment bleaching (Fig. 3) and lipid peroxidation (Fig. 4), there is a question as to how plants could adapt to this stress. In fact, to improve their tolerability to various environmental stresses, plants are able to generate a set of protective and remediation mechanisms for the effective removal of ROS. Among these mechanisms, ROS-eliminating enzymes play an indispensable role and are often used as indicators of abiotic stresses^28,45^.

**Figure 5:**
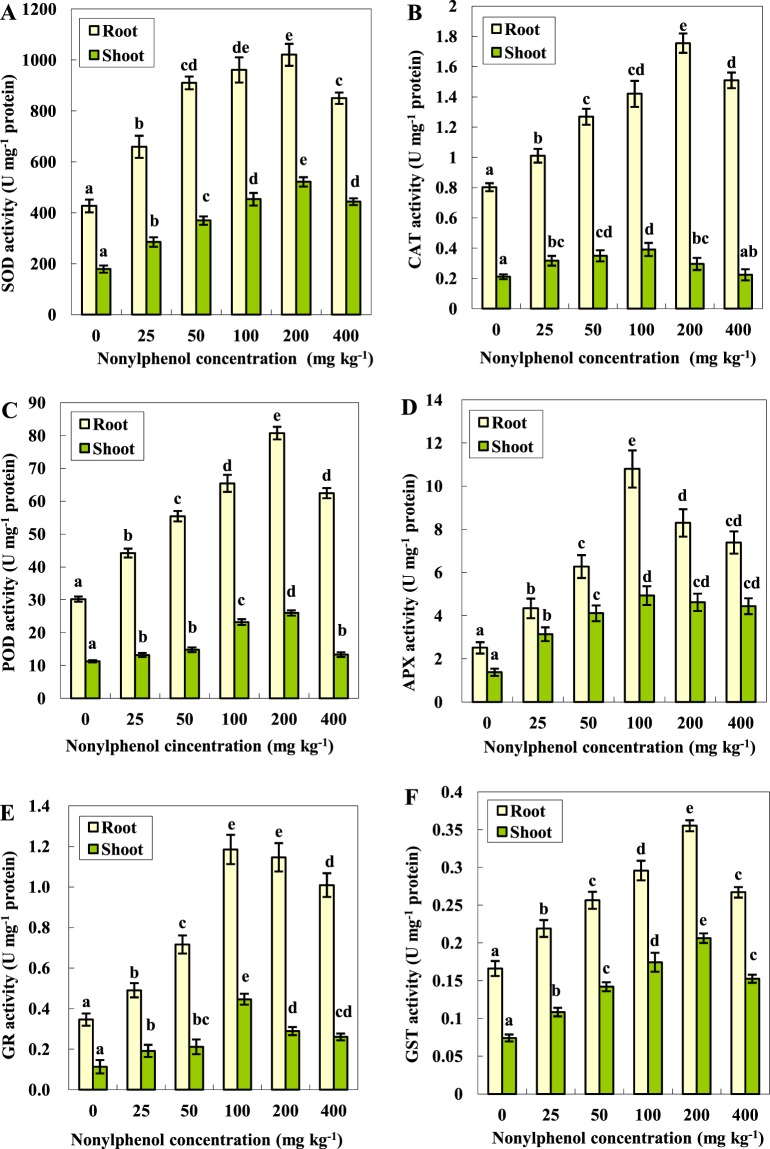
Effect of NP on the activities of SOD (**A**), CAT (**B**), POD (**C**), APX (**D**), GR (**E**) and GST (**F**) in the tomato plants (means ± SD, n = 3). Data with the same letter did not differ significantly at the 5% level.

For plant cells, SOD, which is present in all subcellular structures capable of producing ROS, is regarded as the first barrier to the overproduction of ROS^33,46^. As shown in Fig. 5A, to counteract NP-induced ROS stress, the activity of SOD in both the shoots and roots was dramatically elevated compared with the control (non-NP exposed soil). The peak SOD activity in the shoots and roots occurred concurrently when the NP content of soil was 200 mg kg^−1^ (Fig. 5A).

SOD is responsible for catalysing the disproportionation of O_2_•− to H_2_O_2_ and O_2_, after which the H_2_O_2_ is decomposed into H_2_O and O_2_ by other enzymes such as CAT or POD. With the increase in the soil NP concentration in soil from 25 to 400 mg kg^−1^, the CAT activity experienced a change in its “weak-strong-weak” type, and its peak value in the shoots and roots was observed in the 100 and 200 mg kg^−1^ soil NP treatments at 1.85- and 2.18-fold greater than the control (Fig. 5B). As indicated in Fig. 5C, the POD activity in the shoots and roots of the tomato seedlings was also significantly activated by the NP treatment and synchronously reached its zenith when the NP concentration of the soil was 200 mg kg^−1^, at 2.29- and 2.67-fold greater than the control, respectively. In comparison with the CAT, the POD appeared to be more sensitive to NP stress. The same results were previously reported and attributed to the stronger affinity of POD to H_2_O_2_ than CAT^27,47^.

The AsA-GSH cycle is a major metabolic pathway in plants for the detoxification of H_2_O_2_. It involves antioxidants, namely ascorbate, glutathione and NADPH, as well as enzymes linked to these antioxidants such as APX and GR^48,49^. At the beginning of this cycle, H_2_O_2_ was first converted to H_2_O by APX using ascorbate as the electron donor^50,51^. Davletova *et al*.^52^ previously reported that the APX activity had a close connection to the ROS level in Arabidopsis. Additionally, the data from this study indicated that the APX activity of the tomato seedlings was modulated by NP-induced reactive oxygen stress, or rather that the APX activity could reflect the degree of NP toxicity (Fig. 5D). GR, participates in the final step of the AsA-GSH system to reduce GSSG to GSH in the presence of NADPH, and it was also reportedly related to the conduction of the redox signal^53^. As shown in Fig. 5E, when exposed to 0–400 mg kg^−1^ soil NP, the changes in the GR activity in the shoots and roots were observed to keep pace with the APX every time, which is to say that the AsA-GSH system was fully mobilized to cope with the NP-induced ROS stress^54,55^. Moreover, since GR is responsible for the regeneration of reduced glutathione (GSH) which plays a crucial role in degrading xenobiotics in higher plants^56,57^, the increase in the GR activity in our study was much higher than the increase in the CAT and POD activity, suggesting that the GR activity was elevated to ensure sufficient GSH turnover in response to the NP- induced consumption of GSH. Consistent with our results, Wang *et al*.^24^ found that chlorothalonil exposure increased both the activity and transcript level of GR for the GSH regeneration in tomato leaves.

GST catalyses the covalent binding of various electrophilic xenobiotics to the sulphur atom in GSH, which is a classical detoxification pathway in plant tissues^28,58^. Not long ago, GST was reported to be a potent antidote to atrazine and ametryn in plants^28,30,59^. In addition, our recent study indicated that GST in wheat plants played an important role in the detoxification of simetryne^27^. In the present study, as shown in Fig. 5F, the GST activities in the shoots and roots first kept rising with the increased NP concentration in the soil, until the NP reached 200 mg kg^−1^, and then with the further NP increase of concentration in the soil, the GST activity failed to continue to increase. This phenomenon might be caused by the “stress-elicited oxidative explosion” described by Joo *et al*.^60^ that generated so many ROS, the adaptation and adjustment capacities of the antioxidants in the plant tissues were supassed, thereby inactivating the enzymes^27,61^.

### Transcript abundance

The above results showed that NP-stress could trigger growth depression, loss of chlorophyll, the acceleration of lipid peroxidation and the activation of the antioxidant system. To understand the transcriptional changes that occurred during these processes, we performed a semi-quantitative RT-PCR-based assay on *Cu/Zn-SOD*, *CAT*, *POD*, *APX*, *GST* and *GR*.

**Figure 6:**
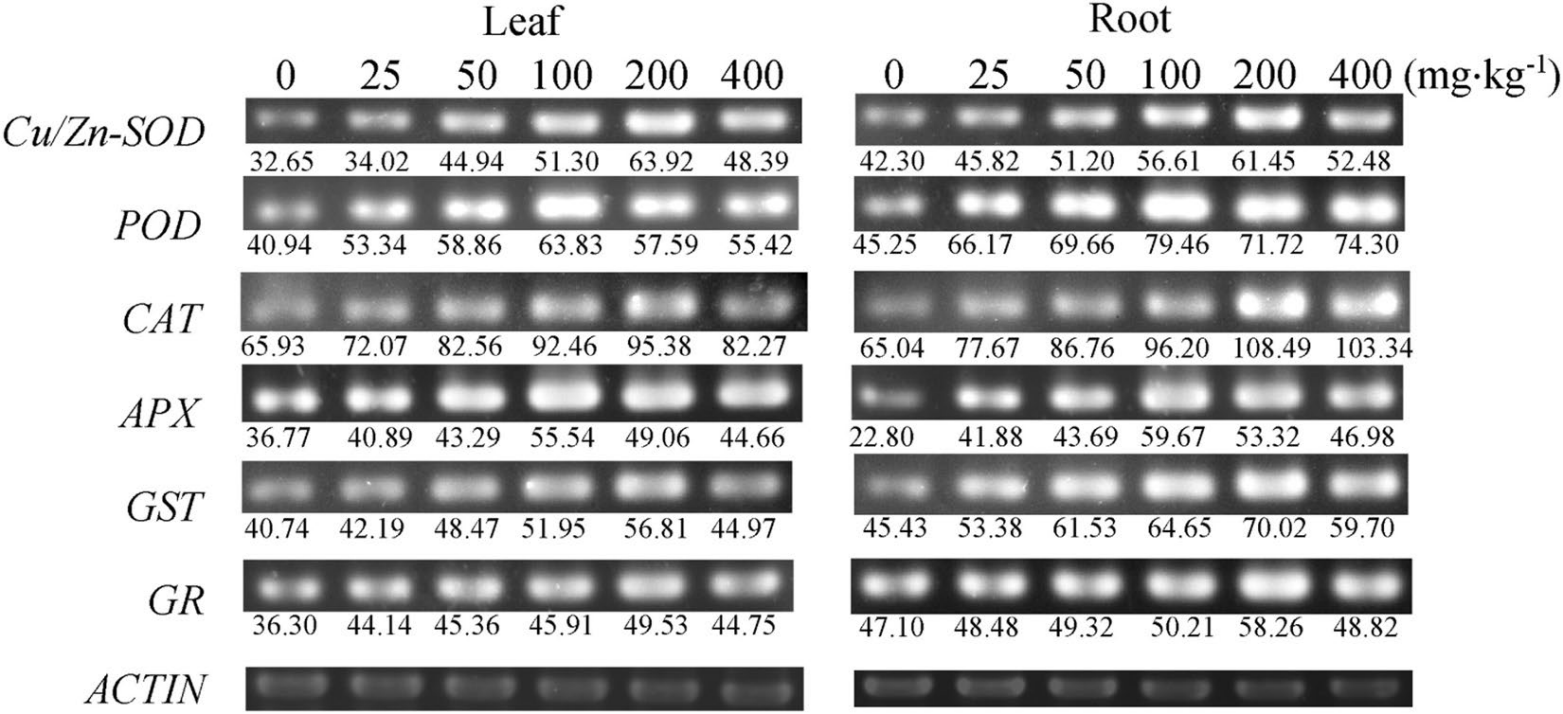
Effects of NP on the transcript abundance of *Cu/Zn-SOD*, *POD*, *CAT*, *APX*, *GST* and *GR* in tomato plants. *ACTIN* was used for cDNA normalization. The PCR products of different genes were loaded to differenrt agarose gels (1%, *w/v*), and the cropped gels were displayed. Images of the full-length PCR gels are presented in Supplementary Fig. 1.

NP treatment resulted in the significant upregulation of *Cu/Zn-SOD* expression in the tomato plants (Fig. 6), and the highest levels in the leaves and roots both occurred at 200 mg kg^−1^ NP, at 2.74-fold and 2.44-fold greater than the control, respectively (Fig. 6). The *POD* and *CAT* expression in the tomato plants was also increased in response to NP exposure, reaching a peak within 100–200 mg kg^−1^ NP. As shown in Fig. 6, the change in the *APX* expression was similar to that of *Cu/Zn-SOD* with a slight difference, in that the maximum expression of *APX* appeared much earlier at 100 mg kg^−1^ NP. This phenomenon suggested that APX might be more sensitive to NP stress than SOD. In addition, the *GST* and *GR* expression shared the same variation pattern with that of *Cu/Zn-SOD*, peaking at 200 mg kg^−1^ NP. Furthermore, by comparing Figs 5 and 6, it is inescapably clear that, apart from the relative abundance of antioxidants in the roots and leaves, the response trend in antioxidants at the transcriptional level was roughly identical to that at the protein level. The same phenomenon was previously reported by Jiang and Yang^36^ and was ascribed to the fact that transcription and translation were two complex mechanisms regulated by many factors, and they were therefore difficult to synchronize completely. Collectively, following NP exposure, the tomato plants comprehensively activated their defence mechanisms at both the molecular and physiological levels to enhance their tolerance to stress. In addition, the transcriptional parameters were good potential molecular markers for indicating the level of genotoxicity induced by exogenous toxicants such as NP.

## Conclusions

The results presented in this study showed that the uptake and accumulation of NP in tomato seedlings became higher with the increased NP concentrations in soil treatments ranging from 25 to 400 mg kg^−1^, which in turn not only intuitively restrained the growth of the seedlings, but it also led to the decline in total chlorophyll and the intensification of membrane lipid peroxidation in the plant tissues. Among the results, the total chlorophyll content of the leaf tissues decreased progressively with the increased soil NP, which would be an efficient indicator of the level of stress caused by NP in the soil-crop system. Moreover, when confronted with NP stress, tomato plants correspondingly evoked their stress-tolerant mechanism. As shown in the present study, the antioxidant system responsible for maintaining the balance between ROS production and clearance in plant tissues was significantly activated via both physiological and biochemical pathways to relieve the NP derived oxidative stress. Given the above findings, the biochemical and molecular responses of plants to NP would not only be useful for indicating the degree of NP contamination in agricultural environments, but it would also be instructive for formulating strategies to address the NP-induced damage to agricultural production, food security and human health.

## Supplementary information

Supplementary Figure 1

## References

[1] Jubendradass R, D’Cruz SC, Rani SJA, Mathur P (2012). Nonylphenol induces apoptosis via mitochondria-and Fas-L-mediated pathways in the liver of adult male rat. Regul. Toxicol. Pharm..

[2] Tyler CR, Jobling S, Sumpter JP (1998). Endocrine disruption in wildlife: a critical review of the evidence. Crit. Rev. Toxicol..

[3] WHO/UNEP (United Nations Environment Programme). *State of the science of endocrine disrupting chemicals-2012* (ed. Bergman, Å.) 1–296 (WHO/UNEP, 2013).

[4] Zala SM, Penn DJ (2004). Abnormal behaviours induced by chemical pollution: a review of the evidence and new challenges. Anim. Behav..

[5] Birks L (2016). Occupational Exposure to Endocrine-Disrupting Chemicals and Birth Weight and Length of Gestation: A European Meta-Analysis. Environ. Health Perspect..

[6] Soares A, Guieysse B, Jefferson B, Cartmell E, Lester JN (2008). Nonylphenol in the environment: a critical review on occurrence, fate, toxicity and treatment in wastewaters. Environ. Int..

[7] Ojeda G (2013). Effects of nonylphenols on soil microbial activity and water retention. Appl. Soil Ecol..

[8] Bechi N (2010). Environmental levels of para-nonylphenol are able to affect cytokine secretion in human placenta. Environ. Health Perspect..

[9] Shan J, Wang YF, Wang LH, Yan XY, Ji R (2014). Effects of the geophagous earthworm Metaphire guillelmi on sorption, mineralization, and bound-residue formation of 4-nonylphenol in an agricultural soil. Environ. Pollut..

[10] Das KC, Xia K (2008). Transformation of 4-nonylphenol isomers during biosolids composting. Chemosphere.

[11] Liu J (2014). Degradation and bound-residue formation of nonylphenol in red soil and the effects of ammonium. Environ. Pollut..

[12] Vogel TM, Gridlle CS, McCarty PL (1987). Transformations of halogenated aliphatic compounds. Environ. Sci. Technol..

[13] Cai QY (2012). Occurrence of nonylphenol and nonylphenol monoethoxylate in soil and vegetables from vegetable farms in the Pearl River Delta, South China. Arch. Environ. Contam. Toxicol..

[14] Jiang L (2018). Effects of earthworm casts on sorption-desorption, degradation, and bioavailability of nonylphenol in soil. Environ. Sci. Pollut. Res..

[15] Roberts P, Roberts JP, Jones DL (2006). Behaviour of the endocrine disrupting chemical nonylphenol in soil: assessing the risk associated with spreading contaminated waste to land. Soil Biol. Biochem..

[16] Vivacqua A (2003). The food contaminants bisphenol A and 4-nonylphenol act as agonists for estrogen receptor alpha in MCF7 breast cancer cells. Endocrine.

[17] Jobling S, Nolan M, Tyler CR, Brighty G, Sumpter JP (1998). Widespread sexual disruption in wild fish. Environ. Sci. Technol..

[18] Bokern M, Raid P, Harms H (1998). Toxicity, uptake and metabolism of 4-n-nonylphenol in root cultures and intact plants under septic and aseptic conditions. Environ. Sci. Pollut. Res..

[19] Sjöström Å, Collins C, Smith S, Shaw G (2008). Degradation and plant uptake of nonylphenol (NP) and nonylphenol-12-ethoxylate (NP12EO) in four contrasting agricultural soils. Environ. Pollut..

[20] Zhang QM (2016). Comparative toxicity of nonylphenol, nonylphenol-4-ethoxylate and nonylphenol-10-ethoxylate to wheat seedlings (*Triticum aestivum* L.). Ecotox. Environ. Safe..

[21] Liu L, Zhen ZW, Luo ZG, Huang BL (2011). Investigation on pollution situation of phenolic substances in the vegetables. J. China Tradit. Chinese medicine Inform. (China).

[22] Murshed R, Lopez-Lauri F, Sallanon H (2014). Effect of salt stress on tomato fruit antioxidant systems depends on fruit development stage. Physiol. Mol. Biol. Plants.

[23] Murshed R, Lopez-Lauri F, Sallanon H (2013). Effect of water stress on antioxidant systems and oxidative parameters in fruits of tomato (Solanum lycopersicon L, cv. Micro-tom). Physiol. Mol. Biol. Plants.

[24] Wang J (2010). The different responses of glutathione-dependent detoxification pathway to fungicide chlorothalonil and carbendazim in tomato leaves. Chemosphere.

[25] Zhou Y (2017). Exogenous glutathione alleviates salt-induced oxidative stress intomato seedlings by regulating glutathione metabolism, redox status,and the antioxidant system. Sci. Hortic-Amsterdam.

[26] Yüzbaşıoğlu E, Dalyan E (2019). Salicylic acid alleviates thiram toxicity by modulating antioxidant enzyme capacity and pesticide detoxification systems in the tomato (*Solanum lycopersicum* Mill.). Plant Physiol. Bioch..

[27] Jiang L (2016). Effects of two different organic amendments addition to soil on sorption–desorption, leaching, bioavailability of penconazole and the growth of wheat (*Triticum aestivum* L.). J. Environ. Manage..

[28] Liu Y (2017). Comprehensive analysis of degradation and accumulation of ametryn in soils and in wheat, maize, ryegrass and alfalfa plants. Ecotoxicol. Environ. safe..

[29] Gill SS, Tuteja N (2010). Reactive oxygen species and antioxidant machinery in abiotic stress tolerance in crop plants. Plant Physiol. Bioch..

[30] Zhang JJ, Lu YC, Zhang JJ, Tan LR, Yang H (2014). Accumulation and toxicological response of atrazine in rice crops. Ecotox. Environ. Safe..

[31] Lichtenthaler, H.K. & Buschmann, C. Chlorophylls and carotenoids: measurement and characterization by UV-VIS spectroscopy. In *Current protocols in food analytical chemistry* (ed. Wrolstad, R. E.) F4.3.1–F4.3.8 (John Wiley and Sons, 2001).

[32] Ohkawa H, Ohishi N, Yagi Y (1979). Assay for lipid peroxides in animal tissue by thiobarbituric acid reaction. Anal. Biochem..

[33] Dai LL (2017). Differential responses of peach (Prunus persica) seedlings to elevated ozone are related with leaf mass per area, antioxidant enzymes activity rather than stomatal conductance. Environ. Pollut..

[34] Castillo FI, Penel I, Greppin H (1984). Peroxidase release induced by ozone in Sedum album leaves. Plant Physiol..

[35] McKee IF, Eiblmeier M, Polle A (1997). Enhanced ozone-tolerance in wheat grown at an elevated CO_2_ concentration: ozone exclusion and detoxification. New Phytol..

[36] Jiang L, Yang H (2009). Prometryne-induced oxidative stress and impact on antioxidant enzymes in wheat. Ecotox. Environ. Safe..

[37] Habig WH, Pabst MJ, Jakoby WB (1974). Glutathione S-transferases. The first enzymatic step in mercapturic acid formation. J. Biol. Chem..

[38] Foyer CH (1995). Over expression of glutathione reductase but not glutathione synthetase leads to increases in antioxidant capacity and resistance to photoinhibition in poplar trees. Plant Physiol..

[39] Lv DZ (2010). Determination of alkylphenols and alkylphenol ethoxylates in tropical fruits by LC with ultrasonic-assisted extraction. Food Sci (China).

[40] Villar-Navarro M, Ramos-Payán M, Fernández-Torres R, Callejón-Mochón M, Bello-López MÁ (2013). A novel application of three phase hollow fiber based liquid phase microextraction (HF-LPME) for the HPLC determination of two endocrine disrupting compounds (EDCs), n-octylphenol and n-nonylphenol, in environmental waters. Sci. Total Environ..

[41] Jiao LY, Ding HZ, Wang LH, Zhou Q, Huang XH (2017). Bisphenol A effects on the chlorophyll contents in soybean at different growth stages. Environ. Pollut..

[42] Qiu Z, Wang L, Zhou Q (2013). Effects of bisphenol A on growth, photosynthesis and chlorophyll fluorescence in above-ground organs of soybean seedlings. Chemosphere.

[43] Mano J (2012). Reactive carbonyl species: Their production from lipid peroxides, action in environmental stress, and the detoxification mechanism. Plant Physiol. Bioch..

[44] Percival GC (2017). The influence of glyphosate on carotenoid pigments, reactive oxygen species scavenging enzymes and secondary stress metabolites within leaf tissue of three Acer species. Urban For. Urban Gree..

[45] Aghababaei F, Raiesi F (2015). Mycorrhizal fungi and earthworms reduce antioxidant enzyme activities in maize and sunflower plants grown in Cd-polluted soils. Soil Bio. Biochem..

[46] Bernard F (2014). Antioxidant responses of annelids, Brassicaceae and Fabaceae to pollutants: a review. Ecotoxicol. Environ. Safe..

[47] Mittler R (2002). Oxidative stress, antioxidants and stress tolerance. Trends Plant Sci..

[48] Foyer CH, Noctor G (2005). Redox homeostasis and antioxidant signaling: a metabolic interface between stress perception and physiological responses. The Plant Cell.

[49] Rogers HJ (2012). Is there an important role for reactive oxygen species and redox regulation during floral senescence?. Plant Cell Environ..

[50] Keunen E, Peshev D, Vangronsveld J, Van Den Ende W, Cuypers A (2013). Plant sugars are crucial players in the oxidative challenge during abiotic stress: extending the traditional concept. Plant, Cell Environ..

[51] Queval G, Jaillard D, Zechmann B, Noctor G (2011). Increased intracellular H_2_O_2_ availability preferentially drives glutathione accumulation in vacuoles and chloroplasts. Plant, Cell Environ..

[52] Davletova S (2005). Cytosolic ascorbate peroxidase 1 is a central component of the reactive oxygen gene network of Arabidopsis. The Plant Cell.

[53] Zechmann B, Stumpe M, Mauch F (2011). Immunocytochemical determination of the subcellular distribution of ascorbate in plants. Planta.

[54] Hong CY (2009). NaCl-induced expression of glutathione reductase in roots of rice (*Oryza sativa* L.) seedlings is mediated through hydrogen peroxide but not abscisic acid. Plant Soil.

[55] Noctor G, Foyer CH (1998). Ascorbate and glutathione: keeping active oxygen under control. Annu. Rev. Plant Physiol. Plant Mol. Biol..

[56] Bártíková H (2015). Xenobiotic-metabolizing enzymes in plants and their role in uptake and biotransformation of veterinary drugs in the environment. Drug Metab. Rev..

[57] Murshed R, Lopez-Lauri F, Sallanon H (2008). Microplate quantification of enzymes of the plant ascorbate-glutathione cycle. Anal. Biochem..

[58] Noctor G (2012). Glutathione in plants: an integrated overview. Plant, Cell Environ..

[59] Lu YC, Zhang JJ, Luo F, Huang MT, Yang H (2016). RNA-sequencing Oryza sativa transcriptome in response to herbicide isoprotruon and characterization of genes involved in IPU detoxification. RSC Adv..

[60] Joo JH, Wang SY, Chen JG, Jones AM, Fedoroff NV (2005). Different signaling and cell death roles of heterotrimeric G protein α and β subunits in the arabidopsis oxidative stress response to ozone. Plant Cell.

[61] Mashoudi S, Chaoui A, Ghorbal MH, Ferjani EE (1997). Response of antioxidant enzymes to excess copper in tomato (*Lycopersicon esculentum*, Mill). Plant Sci..

