# Supplementary material for: Accumulation and toxicological effects of nonylphenol in tomato (Solanum lycopersicum L) plants
Source: Sci Rep. 2019 May 7;9:7022. doi: 10.1038/s41598-019-43550-7 (PMC6504949; doi:10.1038/s41598-019-43550-7)
Supplement: Supplementary file 1 — Supplementary Figure 1 [file 41598_2019_43550_MOESM1_ESM.pdf]

Supplementary information includes whole images for Figure 6.

Accumulation and toxicological effects of nonylphenol in tomato (*Solanum lycopersicum* L) plants

Lei Jiang[a,b #], Yi Yang[a,b #], Yong Zhang[c], Ying Liu[a,b], Bo Pan[a], Bingjie Wang [a], Yong Lin[a*]

Supplementary whole images for Figure 6.

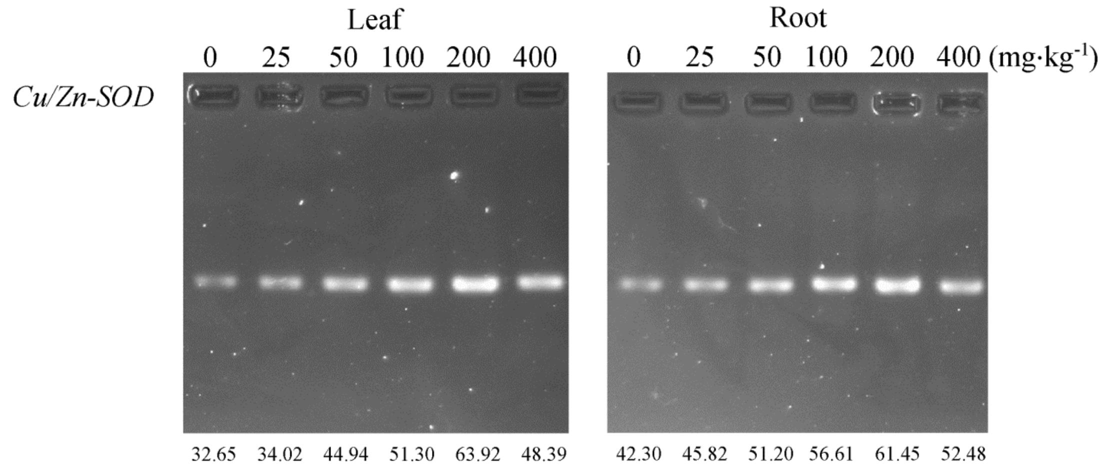

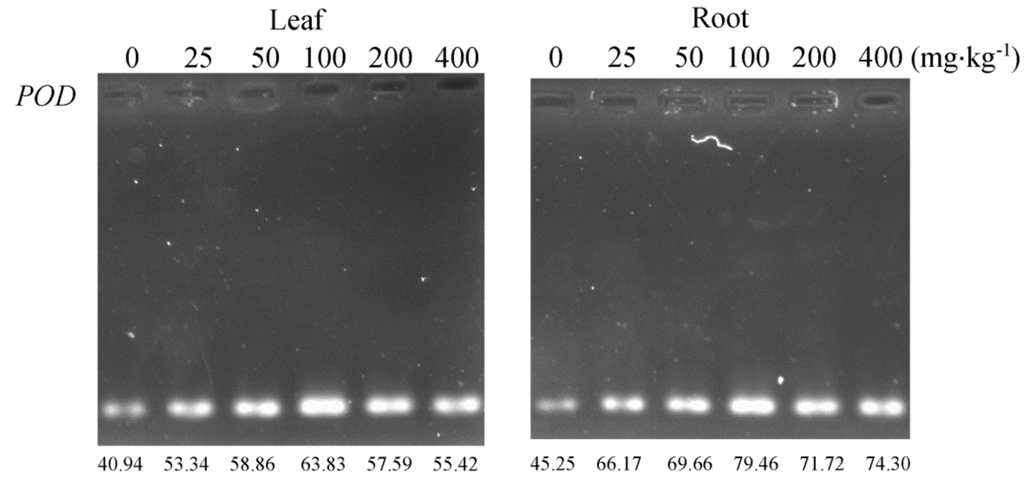

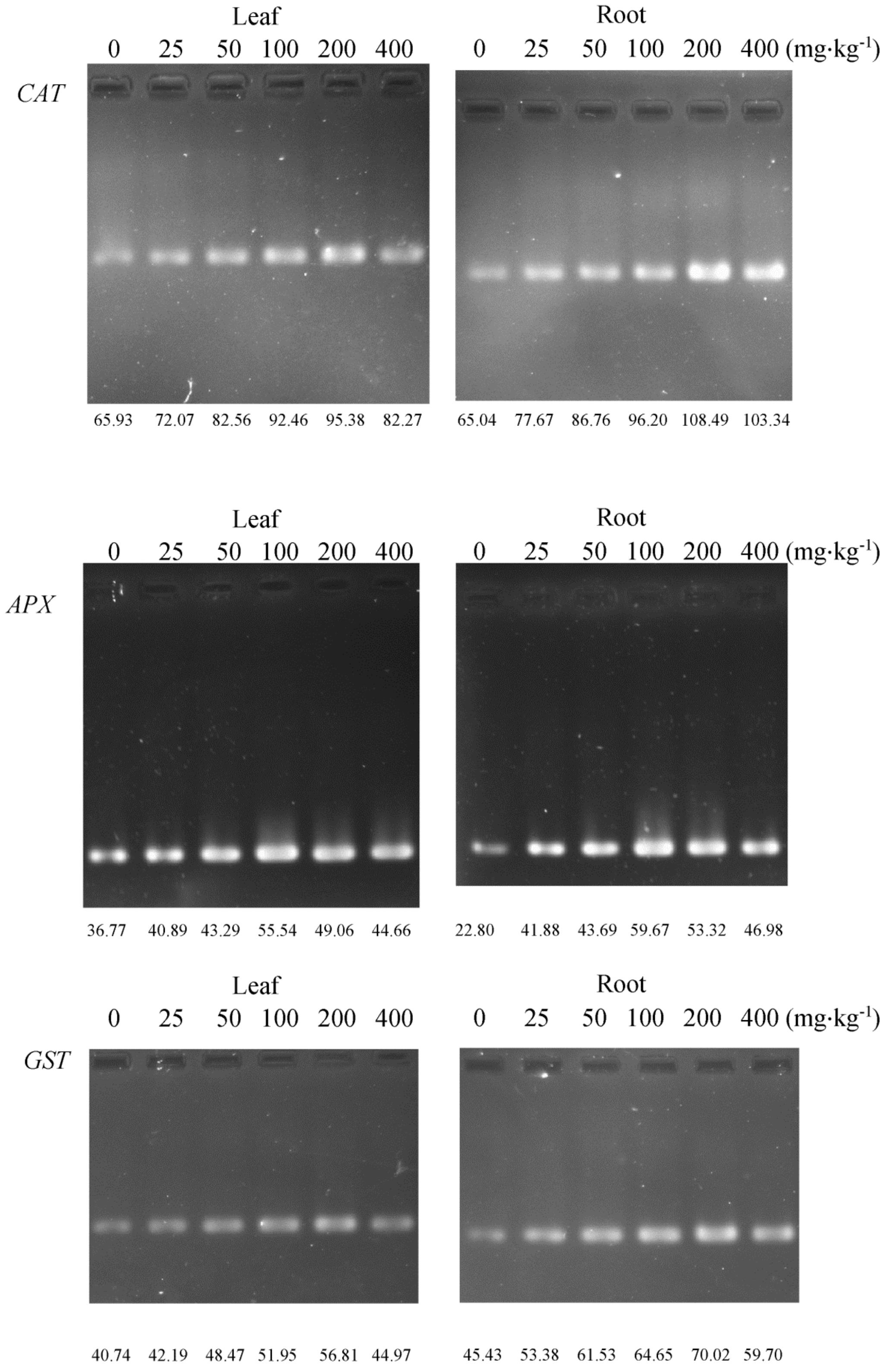
Leaf
Root
0 25 50 100 200 400
0 25 50 100 200 400 (mg·kg-1)
CAT
65.93 72.07 82.56 92.46 95.38 82.27
65.04 77.67 86.76 96.20 108.49 103.34
Leaf
Root
0 25 50 100 200 400
0 25 50 100 200 400 (mg·kg-1)
APX
36.77 40.89 43.29 55.54 49.06 44.66
22.80 41.88 43.69 59.67 53.32 46.98
Leaf
Root
0 25 50 100 200 400
0 25 50 100 200 400 (mg·kg-1)
GST
40.74 42.19 48.47 51.95 56.81 44.97
45.43 53.38 61.53 64.65 70.02 59.70

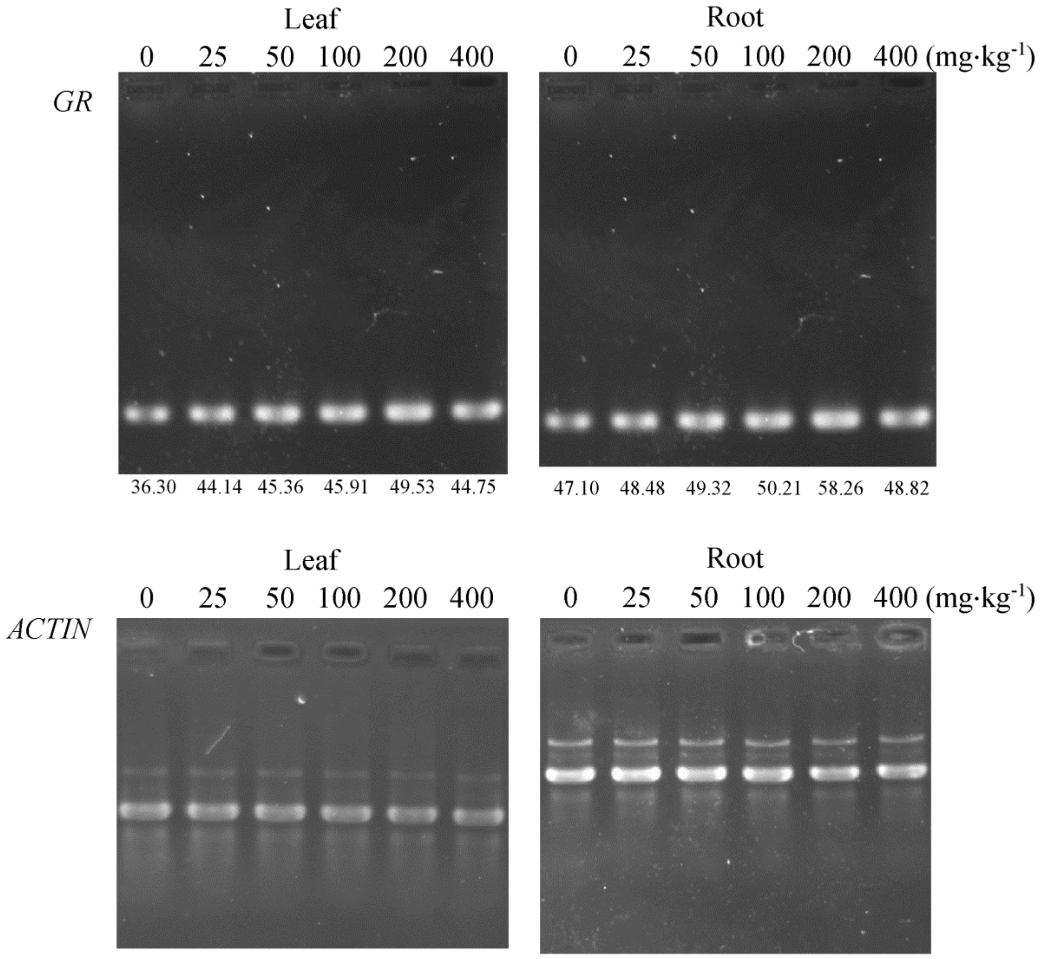

Supplementary Figure 1. Full-length gels for Figure 6.
